# Association of hepatorenal function biomarkers with depression risk in geriatric psychiatry inpatient: a hospital-based cross-sectional study

**DOI:** 10.1186/s12877-025-06536-x

**Published:** 2025-10-31

**Authors:** Shijie Mao, Fanhao Meng, Jianchao Shen, Yu Cheng, Chao Yan, Weimin Zhou

**Affiliations:** 1 Department of Pharmacy, Huzhou Third Municipal Hospital Affiliated to Huzhou University, Huzhou, 313000 China; 2 Department of General Surgery, Huzhou Third Municipal Hospital Affiliated to Huzhou University, Huzhou, 313000 China; 3 Department of Psychiatry, Huzhou Third Municipal Hospital Affiliated to Huzhou University, Huzhou, 313000 China; 4 Department of Urology, Huzhou Third Municipal Hospital Affiliated to Huzhou University, Huzhou, 313000 China; 5 Department of Critical Care Medicine, Huzhou Third Municipal Hospital Affiliated to Huzhou University, Huzhou, 313000 China; 6 Department of Health Information Technology, Huzhou Third Municipal Hospital Affiliated to Huzhou University, Huzhou, 313000 China

**Keywords:** ALT/AST ratio, BUN, BUN/Cr ratio, Geriatric depression

## Abstract

**Background:**

Emerging evidences have suggested a potential link between hepatorenal dysfunction and depression. However, the association between hepatorenal function biomarkers and geriatric depression remains unclear. This study aimed to evaluate the contribution of hepatorenal function biomarkers to depression risk in elderly psychiatric inpatients and identify potential predictive biomarkers for geriatric depression.

**Methods:**

This cross-sectional study analyzed electronic medical records of geriatric psychiatric inpatients (July 2021-December 2024) from a hospital in Eastern China. Multivariable logistic regression models were applied to examine associations between alanine aminotransferase (ALT)/aspartate aminotransferase (AST) ratio, blood urea nitrogen (BUN), BUN/creatinine (Cr) ratio and depression risk, with restricted cubic spline models assessing dose–response relationships. Progressive adjustments included demographic characteristics, metabolic indicators, inflammatory markers, and comorbidities. Subgroup analyses with interaction tests evaluated heterogeneity across predefined subgroups.

**Results:**

Analysis of 1,783 participants (688 with depression) revealed that depressed patients exhibited a higher ALT/AST ratio (0.9 vs. 0.8; *P* < 0.001) with lower BUN (5.4 vs. 6.5 mmol/L; *P* < 0.001) and BUN/Cr ratio (19.4 vs. 21.2; *P* < 0.001). Adjusted models demonstrated that each 1-unit increase in ALT/AST ratio was associated with a 106% higher risk of depression (OR = 2.06, 95% CI: 1.41–3.01; *P* < 0.001), whereas each 1-unit increase in BUN and BUN/Cr ratio corresponded to 16% (OR = 0.84, 95% CI: 0.79–0.89) and 4% (OR = 0.96, 95% CI: 0.94–0.98) decreased risks, respectively (*P* < 0.001). Restricted cubic spline analyses identified linear dose–response relationships: ALT/AST ratio positively correlated with depression risk (*P* < 0.001), while lower levels of BUN (*P* < 0.001) and BUN/Cr ratio (*P* < 0.001) inversely associated with depression risk, with no significant heterogeneity across subgroups (interaction *P* > 0.05).

**Conclusion:**

Hepatorenal function biomarkers (ALT/AST ratio, BUN, BUN/Cr ratio) demonstrate independent associations with geriatric depression risk, suggesting potential involvement of liver-brain and kidney-brain axis in depressive pathophysiology. We recommend incorporating these biomarkers into depression risk stratification protocols for elderly inpatients and propose novel research avenues to elucidate multi-organ crosstalk mechanisms.

## Highlights

1. Hepatorenal biomarkers (ALT/AST ratio, BUN, BUN/Cr ratio) showed independently correlations with geriatric depression risk.

2. Linear associations: ALT/AST ratio positively correlated with depression risk, while BUN levels and BUN/Cr ratio showed inverse correlations.

3. Hepatorenal biomarker abnormalities may reflect liver-brain and kidney-brain crosstalk, highlighting multi-organ pathways in depression and urging clinical integration for early risk detection.

**Graphical abstract:**

## Graphical abstract

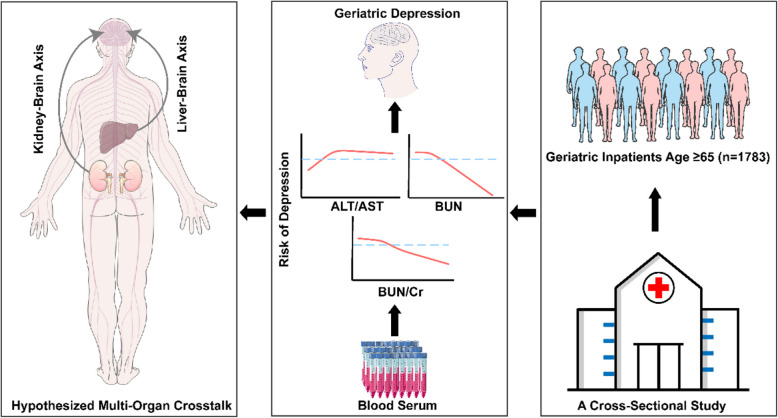

## Introduction

Geriatric depression represents a prevalent psychiatric disorder that substantially compromises quality of life in older adults. Globally, approximately 13.3% of older adults are reported to have major depressive disorder (MDD) [1]. Clinical epidemiology has revealed significantly higher prevalence rates of geriatric depression in healthcare settings versus community-dwelling populations in China (18.1% vs. 11.6%, *P* < 0.001) [2]. Multimorbidity is highly prevalent among older adults with depression. Research interest in the association between chronic health conditions and depression risk has significantly increased over recent decades [3]. WHO epidemiological evidence demonstrates a statistically significant elevation in depression prevalence among individuals with chronic diseases compared to those without (*P* < 0.0001) [4]. Data from the China Health and Retirement Longitudinal Study have demonstrated that individuals with chronic conditions or multimorbidity exhibit increased risk of depression compared to non-chronic counterparts [5, 6].

Chronic liver disease (CLD) and chronic kidney disease (CKD) are prevalent chronic conditions in older adults, with age-dependent prevalence escalation [7, 8]. Compared to the general population (10% depression prevalence), CLD (18–58%) and CKD (20–25%) cohorts exhibit substantially higher depression incidence rates, comparable to the elevated rates observed in other medical comorbidities and chronic inflammatory conditions [9, 10]. Furthermore, significant depression associations persist across CLD subtypes, including metabolic-associated fatty liver disease, alcoholic liver disease, viral hepatitis, and drug-induced liver injury [11]. Bidirectional relationships characterize CKD-depression interactions: CKD elevates depression incidence while depression increases CKD progression risk [12, 13]. In cirrhotic patients, reduced hepatic monoamine oxidase-B activity leads to phenethylamine accumulation in the brain by crossing the blood–brain barrier, thereby activating microglia and triggering astrocyte swelling with consequent cognitive impairment [14]. The hypothalamic–pituitary–adrenal (HPA) axis is recognized as a critical biological pathway in depression, modulating the body's stress response. Chronic stress induces hyperactivation of the HPA axis, elevating cortisol levels which subsequently cause neuronal damage, particularly in the hippocampus and prefrontal cortex—regions intrinsically linked to emotion regulation and psychiatric disorders [15]. Recent studies have established significant associations between depressive symptoms and hepatic biomarkers (γ-glutamyltransferase [GGT], alanine aminotransferase [ALT], aspartate aminotransferase [AST], AST/ALT ratio), renal biomarkers (creatinine [Cr], blood urea nitrogen [BUN]), and inflammatory biomarkers (tumor necrosis factor-α [TNF-α], interleukin-6 [IL-6], high-sensitivity C-reactive protein [hs-CRP]) [16, 17]. Studies indicate that inflammatory markers (e.g., CRP, IL-6) and liver enzyme ratios (e.g., AST/ALT ratio) significantly predict emotional symptom scores [16]. Furthermore, BUN levels show a significant association with depression, potentially mediated by accelerating atherosclerosis in chronic dialysis patients and impairing the medial prefrontal cortex, thereby contributing to depressive pathogenesis [18]. Sustained increases in Cr and BUN may also induce white matter lesions, disrupting information processing across brain regions [19]. Consequently, further exploration of the relationship between hepatorenal biomarkers and depression risk is critical. However, current research predominantly focused on hepatic/renal dysfunction risks in depressed populations, while the association between hepatorenal insufficiency and geriatric depression remains underexplored. Most studies lack real-world data and inadequately adjust for medication confounders (e.g., hepatoprotectant use obscuring hepatic dysfunction), compromising result validity.

Based on the existing "hepato-reno-cerebral axis" hypothesis of multi-organ interaction, this cross-sectional study investigates the associations between routinely measured hepatorenal biomarkers (ALT/AST ratio, BUN and BUN/Cr ratio) and depression risk in geriatric psychiatry inpatients, to validate our hypothesis that these markers are independent risk factors for geriatric depression onset. These biomarkers were selected based on their established clinical relevance: the ALT/AST ratio serves as a critical indicator of hepatic metabolic dysfunction and fibrosis [20–22], while the BUN/Cr ratio is a sensitive marker of renal insufficiency [23]. Ultimately, a predictive stratification tool for geriatric depression based on routine biochemical indicators will be built to provide a low-cost screening protocol for resource-limited clinical settings, facilitating the implementation of early warning systems and targeted interventions.

## Materials and method

### Data source and study population

This cross-sectional study analyzed electronic medical records of all geriatric psychiatry inpatients admitted to Huzhou Third Municipal Hospital between July 2020 and December 2024 (*n =* 4,445). Data extraction excluded all personal identifiers, retaining only clinical variables. The Ethics Committee of Huzhou Third Municipal Hospital approved this low-risk retrospective observational study (Approval No. 2025–119). Clinical trial number: not applicable.

**Fig. 1:**
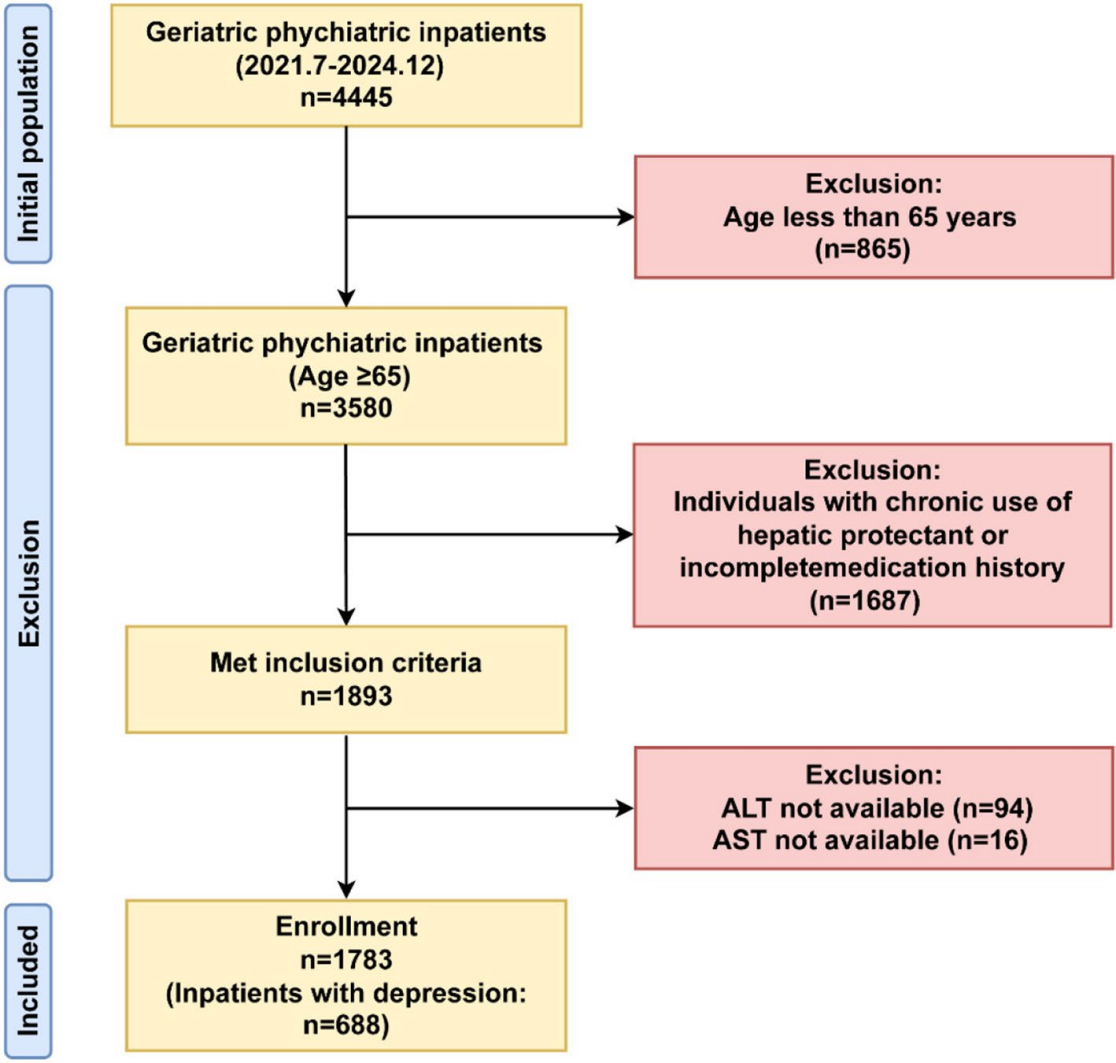
Flow chart of the study population inclusion

Eligibility required complete documentation of pre-admission medications and hepatorenal function biomarker panels (ALT, AST, BUN, Cr). Patients using hepatoprotective agents were excluded to avoid underestimation of liver injury biomarkers (ALT/AST ratio). Hepatoprotective agents may normalize ALT/AST ratio levels in individuals with subclinical hepatic impairment, thereby obscuring the true association between liver function biomarker (ALT/AST ratio) and depression risk. However, this restriction may limit generalizability to elderly populations actively managed with such medications. After excluding patients with hepatoprotectant exposure or incomplete data, the final analytical sample included 1,783 patients. Figure 1 illustrates a schematic of the selection methodology.

### Hepatorenal function biomarkers

The primary independent variables were ALT/AST ratio, BUN (mmol/L), and BUN/Cr ratio. All biomarkers were measured within 24 h of admission using chemiluminescence microparticle immunoassay (CMIA) on an Abbott Architect i2000 analyzer (Abbott Diagnostics, Chicago, USA). The proteinuria was assessed by dipstick test. Ratios were calculated as: (1) ALT (U/L)/AST (U/L) [24], (2) BUN (mg/dL)/Cr (mg/dL) [25]. Variables were categorized into quartiles for analysis.

### Assessment of depression

Depression diagnosis required meeting DSM-5 criteria [26]:

- MDD: ≥ 5 core symptoms (including depressed mood/anhedonia) over ≥ 2 weeks.
- Persistent Depressive Disorder: Depressive symptoms lasting ≥ 2 years with ≤ 2 months' remission.
- Standardized assessments included MINI 7.0 [27] or SCID-5 [28], confirmed by psychiatrists.

### Covariates

Demographic/clinical covariates: age, sex, education, personality, marital status, smoking, drinking, viral hepatitis (VH), fatty liver (FL), cerebral infarction (CI), anxiety, schizophrenia, bipolar disorder, blood pressure (systolic blood pressure [SBP]/diastolic blood pressure [DBP], mmHg), glucose (GLU, mmol/L), lipids (triglyceride [TG]/total cholesterol [TC], mmol/L), albumin (ALB, g/L), prothrombin time (PT, s), white blood cell (WBC, 10⁹/L), total bilirubin (TBIL, μmol/L), eGFR (mL/min/1.73m^2^), proteinuria, GGT/ALT/AST/alkaline phosphatase (ALP) (U/L), BUN (mmol/L), Cr (μmol/L) and antipsychotic drugs using.

Missing values in covariates were addressed using a multivariate single imputation method. This approach utilized an iterative imputer, with a Bayesian Ridge model serving as the estimator in each step of the round-robin imputation process. The validity of this method is supported by evidence that when covariates exhibit ≤ 10% missingness, single imputation negligibly impacts statistical power and effect estimation [29].

### Statistical analysis

In our primary analysis of datasets, continuous variables with normal distribution were described using mean ± standard deviation (SD), skewed continuous variables using median (interquartile range, IQR), and categorical variables as frequencies (percentages). Between-group comparisons utilized: independent Student's t-tests (normal continuous data), Mann–Whitney U tests (non-normal continuous data), and χ^2^ tests (categorical variables). Logistic regression models generated odds ratios (ORs) with 95% confidence intervals (CIs).

To investigate the association between biomarkers and depression risk, we categorized ALT/AST ratio, BUN and BUN/Cr ratio into quartiles (Q1-Q4) based on clinical thresholds. Three progressively adjusted models were developed: Model 1 controlled for age and sex; Model 2 added lifestyle factors (smoking, drinking) and sociodemographics (personality, marital status, education); Model 3 further incorporated biological markers (blood pressure, GLU, lipids, ALB, WBC, eGFR and proteinuria), chronic comorbidities (VH, FL, CI), psychiatric comorbidities (anxiety, schizophrenia, bipolar disorder) and antipsychotic drugs using, thus comprehensively accounting for confounders. The robustness of study outcomes was verified by comparing effect magnitudes and *p*-values across the three refined models. This approach ensured the reliability of conclusions through systematic evaluation of potential influences on findings. Subsequently, we applied restricted cubic spline (RCS) models with four knots (5th, 35th, 65th, 95th percentiles) to evaluate relationships between these biomarkers and depression risk, adjusting for covariates as specified in Model 3. Covariate selection adhered to predefined criteria: clinical relevance, prior literature evidence, statistical significance in univariate analyses (*P* < 0.05), or variables altering effect estimates by > 10%.

Interaction and stratified analyses were performed across prespecified subgroups defined by age (< 80 vs. ≥ 80 years), sex, education, personality, drinking status, FL and CI. Interaction effects between ALT/AST ratio, BUN and BUN/Cr ratio with subgroup variables were evaluated using likelihood ratio tests. Moreover, sensitivity analyses were conducted employing a complete-case analysis strategy.

The statistical analyses in the study were carried out using R Statistical Software (Version 4.2.2, http://www.R-project.org, The R Foundation) and the Free Statistics Analysis Platform (Version 1.9, http://www.clinicalscientists.cn/freestatistics). A two-sided *P* < 0.05 was considered statistically significant.

## Results

### Baseline characteristic of participants

The final analytical cohort comprised 1,783 participants, with 688 (38.6%) in the depression group and 1,095 (61.4%) in the non-depression group. Table 1 presents baseline characteristics demonstrating significant between-group heterogeneity (*P* < 0.05) across demographic, clinical, and laboratory parameters. The depression group exhibited higher female predominance (71.4% vs. 28.6%), younger mean age (71.3 ± 6.4 vs. 73.7 ± 7.6 years), and distinct personality trait distributions (*P* < 0.001). Clinically, depression patients showed elevated prevalence of viral hepatitis (3.49% vs. 0.9%) and fatty liver (18.9% vs. 12.4%; both *P* < 0.001).

**Table 1:** Baseline characteristics among individuals with non-depression and depression

Characteristic	Total	Non-depression	Depression	*P-value*
	**(*n =* 1783)**	**0 (*n =* 1095)**	**1 (*n =* 688)**	
Sex, n (%)				< 0.001
Male	687 (38.5)	490 (44.7)	197 (28.6)	
Female	1096 (61.5)	605 (55.3)	491 (71.4)	
Age (year), Mean ± SD	73.4 ± 7.5	73.7 ± 7.6	71.3 ± 6.4	< 0.001
Marital, n (%)				0.034
Unmarried	56 (3.1)	42 (3.8)	14 (2)	
Married	1727 (96.9)	1053 (96.2)	674 (98)	
Personality, n (%)				< 0.001
Introverted	198 (11.1)	154 (14.1)	44 (6.4)	
Extraverted	1154 (64.7)	699 (63.8)	455 (66.1)	
Other	431 (24.2)	242 (22.1)	189 (27.5)	
Education, n (%)				0.411
No	609 (34.2)	366 (33.4)	243 (35.3)	
Yes	1174 (65.8)	729 (66.6)	445 (64.7)	
Personality, n (%)				< 0.001
Introverted	198 (11.1)	154 (14.1)	44 (6.4)	
Extroverted	1154 (64.7)	699 (63.8)	455 (66.1)	
Other	431 (24.2)	242 (22.1)	189 (27.5)	
Drinking, n (%)				0.005
Never	1525 (85.9)	913 (83.8)	612 (89.2)	
Former	95 (5.3)	65 (6)	30 (4.4)	
Current	156 (8.8)	112 (10.3)	44 (6.4)	
Smoking, n (%)				0.888
Never	1521 (85.4)	931 (85.3)	590 (85.8)	
Former	107 (6.0)	65 (6)	42 (6.1)	
Current	152 (8.5)	96 (8.8)	56 (8.1)	
VH, n (%)				< 0.001
No	1749 (98.1)	1085 (99.1)	664 (96.5)	
Yes	34 (1.9)	10 (0.9)	24 (3.5)	
FL, n (%)				< 0.001
No	1517 (85.1)	959 (87.6)	558 (81.1)	
Yes	266 (14.9)	136 (12.4)	130 (18.9)	
CI, n (%)				0.864
No	1305 (73.2)	803 (73.3)	502 (73)	
Yes	478 (26.8)	292 (26.7)	186 (27)	
Schizophrenia, n (%)				< 0.001
No	1648 (92.4)	960 (87.7)	688 (100)	
Yes	135 (7.6)	135 (12.3)	0 (0)	
Anxiety, n (%)				< 0.001
No	1736 (97.4)	1048 (95.7)	688 (100)	
Yes	47 (2.6)	47 (4.3)	0 (0)	
Bipolar disorder, n (%)				< 0.001
No	1708 (95.8)	1020 (93.2)	688 (100)	
Yes	75 (4.2)	75 (6.8)	0 (0)	
Antipsychotic drugs using, n (%)				< 0.001
No	1182 (66.3)	635 (58)	547 (79.5)	
Yes	601 (33.7)	460 (42)	141 (20.5)	
SBP (mmHg), Mean ± SD	136.1 ± 19.9	135.8 ± 20.5	136.5 ± 18.9	0.515
DBP (mmHg), Mean ± SD	77.2 ± 11.6	76.4 ± 11.8	78.5 ± 11.0	< 0.001
GLU (mmol/L), Mean ± SD	6.4 ± 2.6	6.5 ± 2.8	6.3 ± 2.2	0.214
TG (mmol/L), Median (IQR)	1.2 (0.9, 1.7)	1.1 (0.9, 1.6)	1.3 (1.0, 1.9)	< 0.001
TC (mmol/L), Mean ± SD	4.6 ± 1.2	4.4 ± 1.2	4.8 ± 1.1	< 0.001
ALB (g/L), Mean ± SD	38.7 ± 4.1	38.2 ± 4.3	39.5 ± 3.6	< 0.001
WBC (10⁹/L), Mean ± SD	6.2 ± 2.4	6.2 ± 2.4	6.1 ± 2.5	0.455
PT (s), Mean ± SD	11.6 ± 1.5	11.7 ± 1.3	11.5 ± 1.7	< 0.001
TBIL (μmol/L), Mean ± SD	13.4 ± 6.5	13.7 ± 7.0	12.9 ± 5.4	0.014
GGT (U/L), Median (IQR)	20.1 (14.2, 32.3)	19.9 (13.9, 30.7)	20.7 (14.4, 34.2)	0.078
ALP (U/L), Mean ± SD	82.2 ± 40.1	84.0 ± 46.3	79.5 ± 27.2	0.023
ALT (U/L), Median (IQR)	16.3 (11.7, 24.3)	16.3 (11.9, 24.4)	16.1 (11.4, 24.1)	0.488
AST (U/L), Median (IQR)	21.4 (17.4, 28.6)	22.4 (17.9, 30.1)	20.0 (16.7, 25.8)	< 0.001
Cr (μmol/L), Mean ± SD	78.5 ± 43.9	82.5 ± 51.7	72.0 ± 26.2	< 0.001
eGFR (mL/min/1.73m^2^) Mean ± SD	111.9 ± 43.2	108.6 ± 45.9	117.1 ± 37.8	< 0.001
Proteinuria, n (%)				0.066
Negative/±	1700 (95.3)	1034 (94.4)	666 (96.8)	
1 +	39 (2.2)	28 (2.6)	11 (1.6)	
2 + to 4 +	44 (2.5)	33 (3)	11 (1.6)	
BUN (mmol/L), Mean ± SD	6.1 ± 3.2	6.5 ± 3.7	5.4 ± 2.0	< 0.001
ALT/AST ratio, Mean ± SD	0.8 ± 0.3	0.8 ± 0.3	0.9 ± 0.3	< 0.001
BUN/CR ratio, Mean ± SD	20.5 ± 11.8	21.2 ± 14.2	19.4 ± 6.4	0.002

*Abbreviations*: *VH* Viral hepatitis, *FL* Fatty liver, *CI* Cerebral infarction, *SBP* Systolic blood pressure, *DBP* Diastolic blood pressure, *GLU* Glucose, *TG* Triglyceride, *TC* Total cholesterol, *ALB* Albumin, *WBC* White blood cell, *PT* Prothrombin time, *TBIL* Total bilirubin, *GGT* γ-glutamyl transferase, *ALP* Alkaline phosphatase, *ALT* Alanine aminotransferase, *AST* Aspartate aminotransferase, *Cr* Creatinine, *BUN* Blood urea nitrogen

Laboratory analyses revealed significantly lower diastolic blood pressure (76.4 ± 11.8 vs. 78.5 ± 11.0 mmHg) and renal function markers (creatinine: 72.0 ± 26.2 vs. 82.5 ± 51.7 μmol/L; BUN: 5.4 ± 2.0 vs. 6.5 ± 3.7 mmol/L) in depression patients, contrasted with elevated lipid profiles (triglycerides median: 1.3 vs.1.1 mmol/L; total cholesterol: 4.8 ± 1.1 vs. 4.4 ± 1.2 mmol/L) and albumin levels (39.5 ± 3.6 vs. 38.2 ± 4.3 g/L; all *P* < 0.001). Hepatic parameters showed shortened prothrombin time (11.5 ± 1.7 vs. 11.7 ± 1.3 s) and reduced AST median values (20.0 vs. 22.4 U/L) in depression patients. No significant intergroup differences emerged in education level, smoking history, or cerebral infarction prevalence.

### The association of ALT/AST ratio, BUN and BUN/Cr ratio with depression

Multivariable logistic regression analyses revealed that elevated ALT/AST ratios remained significantly associated with increased depression risk after full covariate adjustment (Model 3, Table 2), demonstrating a 106% risk elevation per unit increase (OR = 2.06, 95% CI: 1.41–3.01, *P* < 0.001). Conversely, ​​elevated BUN (OR = 0.84, 95% CI: 0.79–0.89, *P* < 0.001) and BUN/Cr ratio (OR = 0.96, 95% CI: 0.94–0.98, *P* < 0.001) demonstrated a 16% and 4% reduction in depression risk per unit increase, respectively.

**Table 2:** Association between ALT/AST ratio, BUN and BUN/Cr ratio with depression risk in different models

Variable	Number		Unadjusted		Model 1^a^		Model 2^b^		Model 3^c^	
	Total	Event (%)	OR (95%CI)	***P*** value	OR (95%CI)	***P*** value	OR (95%CI)	***P*** value	OR (95%CI)	***P*** value
ALT/AST ratio	1783	688 (38.6)	1.96 (1.48 ~ 2.6)	< 0.001	1.71 (1.26 ~ 2.3)	< 0.001	1.78 (1.31 ~ 2.41)	< 0.001	2.06 (1.41 ~ 3.01)	< 0.001
Q1 (0.087–0.586)	446	129 (28.9)	1 (Ref)		1 (Ref)		1 (Ref)		1 (Ref)	
Q2 (0.586–0.744)	445	165 (37.1)	1.45 (1.09 ~ 1.92)	0.01	1.27 (0.95 ~ 1.7)	0.104	1.31 (0.97 ~ 1.76)	0.076	1.51 (1.08 ~ 2.12)	0.01
Q3 (0.744–0.963)	446	196 (43.9)	1.93 (1.46 ~ 2.54)	< 0.001	1.71 (1.29 ~ 2.29)	< 0.001	1.79 (1.34 ~ 2.4)	< 0.001	1.97 (1.4 ~ 2.77)	< 0.001
Q4 (0.963–2.870)	446	198 (44.4)	1.96 (1.49 ~ 2.59)	< 0.001	1.7 (1.26 ~ 2.28)	< 0.001	1.77 (1.31 ~ 2.39)	< 0.001	2 (1.4 ~ 2.86)	< 0.001
Trend test				< 0.001		< 0.001		< 0.001		< 0.001
BUN	1783	688 (38.6)	0.86 (0.83 ~ 0.9)	< 0.001	0.89 (0.86 ~ 0.93)	< 0.001	0.89 (0.85 ~ 0.93)	< 0.001	0.84 (0.79 ~ 0.89)	< 0.001
Q1 (1.45–4.26)	446	200 (45.1)	1 (Ref)		1 (Ref)		1 (Ref)		1 (Ref)	
Q2 (4.26–5.41)	445	195 (43.5)	0.94 (0.72 ~ 1.22)	0.627	0.92 (0.7 ~ 1.21)	0.572	0.91 (0.69 ~ 1.2)	0.001	0.88 (0.63 ~ 1.23)	0.451
Q3 (5.41–6.97)	446	173 (38.9)	0.77 (0.59 ~ 1.01)	0.059	0.84 (0.64 ~ 1.1)	0.205	0.83 (0.62 ~ 1.09)	< 0.001	0.75 (0.54 ~ 1.06)	0.1
Q4 (6.97–40.36)	446	120 (26.8)	0.45 (0.34 ~ 0.59)	< 0.001	0.55 (0.41 ~ 0.74)	< 0.001	0.52 (0.39 ~ 0.71)	< 0.001	0.43 (0.29 ~ 0.63)	< 0.001
Trend test				< 0.001		< 0.001		< 0.001		< 0.001
BUN/Cr ratio	1783	688 (38.6)	0.98 (0.96 ~ 0.99)	< 0.001	0.96 (0.95 ~ 0.98)	< 0.001	0.96 (0.95 ~ 0.98)	< 0.001	0.96 (0.94 ~ 0.98)	< 0.001
Q1 (1.73–15.02)	446	180 (40.4)	1 (Ref)		1 (Ref)		1 (Ref)		1 (Ref)	
Q2 (15.02–18.98)	445	192 (43.1)	1.12 (0.86 ~ 1.46)	0.399	1.04 (0.79 ~ 1.37)	0.798	1.05 (0.79 ~ 1.39)	0.737	1.04 (0.75 ~ 1.45)	0.818
Q3 (18.98–23.92)	446	165 (37)	0.87 (0.66 ~ 1.14)	0.303	0.69 (0.52 ~ 0.92)	0.01	0.68 (0.51 ~ 0.91)	0.01	0.66 (0.47 ~ 0.93)	0.017
Q4 (23.92–247.52)	446	151 (33.9)	0.76 (0.58 ~ 0.99)	0.045	0.58 (0.44 ~ 0.78)	< 0.001	0.56 (0.42 ~ 0.75)	< 0.001	0.55 (0.39 ~ 0.79)	0.001
Trend test				0.013		< 0.001		< 0.001		< 0.001

*Abbreviations*: A*LT* Alanine aminotransferase, *AST* Aspartate aminotransferase, *Cr* Creatinine, *BUN* Blood urea nitrogen, *OR* Odds ratios, *CI* Confidence intervals

^a^Adjusted for sex, age

^b^Adjusted for sex, age, smoking, drinking, marital status, personality and education

^c^Adjusted for sex, age, smoking, drinking, marital status, personality, education, systolic blood pressure, diastolic blood pressure, glucose, triglyceride, total cholesterol, albumin, white blood cell, eGFR, proteinuria, viral hepatitis, fatty liver, cerebral infarction, anxiety, schizophrenia, bipolar disorder and antipsychotic drugs using

Unadjusted analyses demonstrated a positive association between ALT/AST ratio and depression when categorized by quartiles (Q4 vs. Q1: OR = 1.96, 95% CI: 1.49–2.59, *P* < 0.001), persisting after adjustment (adjusted OR = 2, 95% CI: 1.4–2.86, *P* < 0.001). BUN (Q4 vs. Q1: OR = 0.45, 95% CI: 0.34–0.59) and BUN/Cr ratio (Q4 vs. Q1: OR = 0.76, 95% CI: 0.58–0.99) exhibited inverse relationships, maintaining significance post-adjustment (BUN: OR = 0.43, 95% CI: 0.29–0.63; BUN/Cr ratio: OR = 0.55, 95% CI: 0.39–0.79; both *P* < 0.001).

**Fig. 2:**
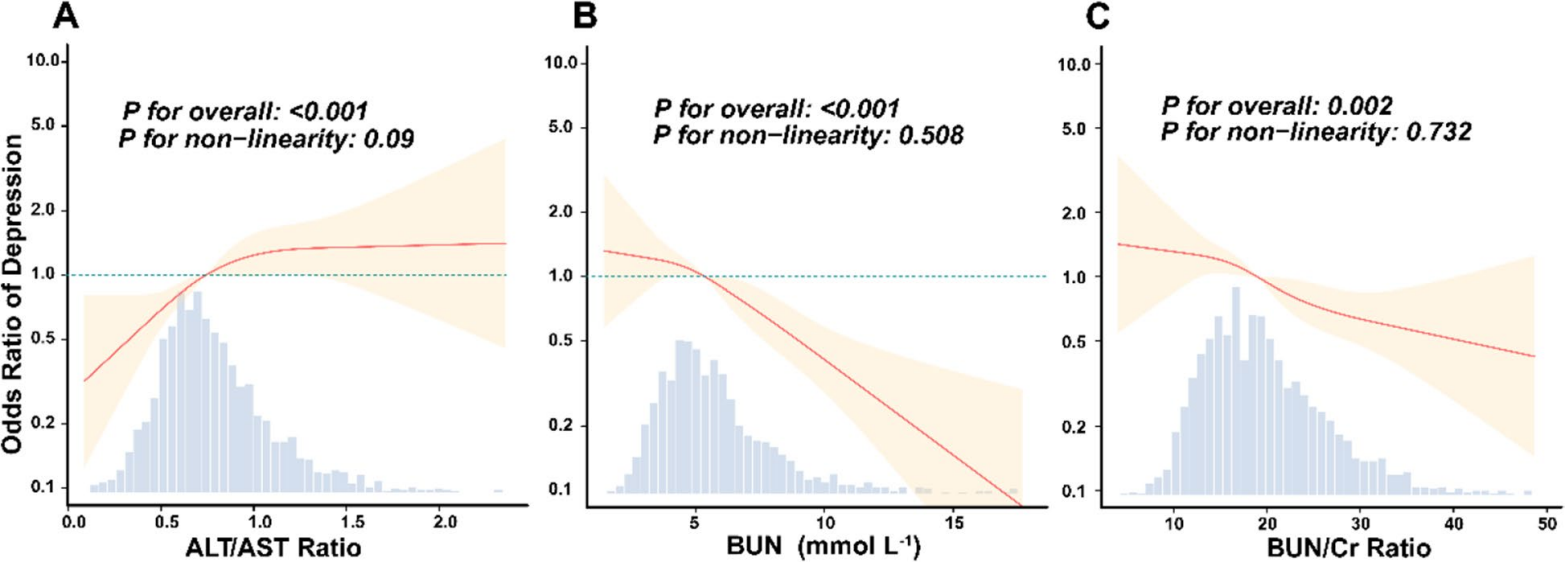
Association between hepatorenal function biomarkers and depression in geriatric psychiatry patients. Data were analyzed using a multivariable logistic regression model incorporating restricted cubic splines, with ALT/AST ratio (**A**), BUN (**B**), and BUN/Cr ratio (**C**) treated as continuous variables. Adjustments were made for sex, age, smoking, drinking, marital status, personality, education, systolic blood pressure, diastolic blood pressure, glucose, triglyceride, total cholesterol, albumin, white blood cell, eGFR, proteinuria, viral hepatitis, fatty liver, cerebral infarction, anxiety, schizophrenia, bipolar disorder and antipsychotic drugs using. ALT, alanine aminotransferase; AST, aspartate aminotransferase; BUN, blood urea nitrogen; Cr, creatinine

Restricted cubic spline analyses demonstrated a significant linear association between elevated ALT/AST ratio and increased depression risk (*P* < 0.001) (Fig. 2A). Conversely, inverse linear correlations were observed for both BUN (*P* < 0.001) and BUN/Cr ratio (*P* < 0.001), with depression risk progressively escalating as biomarker levels decreased (Fig. 2B and C).

### Subgroup analysis and sensitivity analysis

**Fig. 3:**
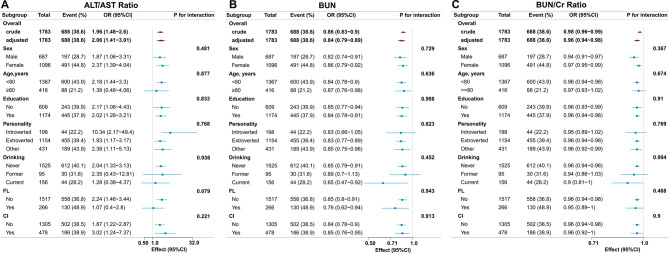
Subgroup analysis of the association between ALT/AST ratio, BUN, BUN/Cr ratio and the incidence of depression. ALT, alanine aminotransferase; AST, aspartate aminotransferase; BUN, blood urea nitrogen; Cr, creatinine. Statistically significant (*P* < 0.05)

To investigate population-specific variations in hepatorenal function biomarker-depression associations, we conducted stratified analyses by age, sex, education, marital status, smoking status, and cerebral infarction history. Forest plots were employed to assess subgroup heterogeneity in the associations of ALT/AST ratio, BUN and BUN/Cr ratio with depression risk (Fig. 3). No statistically significant interaction effects were observed for any biomarkers (all interaction *P*-values > 0.05), indicating consistent associations across demographic and clinical strata. Sensitivity analyses demonstrated no material differences in characteristics between included participants and those excluded due to missing data.

## Discussion

This study establishes hepatorenal dysfunction as an independent risk dimension for geriatric depression through multi-organ biomarker profiling. We demonstrate persistent linear dose–response relationships between ALT/AST ratio, BUN or BUN/Cr ratio levels, and depression risk in elderly inpatients, remaining robust after rigorous adjustment for metabolic-inflammatory confounders. Our findings address three critical limitations in existing literature: 1) Previous single-system approaches focused on isolated hepatic (e.g., ALT) or renal (e.g., BUN) biomarkers [18, 30], while our multi-system assessment quantifies cumulative hepatorenal effects; 2) Most studies failed to exclude hepatoprotectant users, potentially masking hepatic dysfunction-depression associations; 3) We incorporated understudied confounders including metabolic disorders (GLU/lipid profiles), systemic inflammation (WBC), comorbidities (VH, FL, CI, anxiety, schizophrenia, bipolar disorder) and antipsychotic drugs using, confirming the biological independence of observed associations from conventional risk factors.

The observed association may reflect intertwined organ-brain communication mechanisms underlying depression pathogenesis [31]. Clinical studies have documented elevated cerebral ammonia levels in patients with hepatic dysfunction [32]. Impaired hepatic ammonia clearance precipitates hyperammonemia, which facilitates neuropsychiatric manifestations through ammonia accumulation in the central nervous system [33, 34]. Hyperammonemia impair glutamate processing in the hippocampus by disrupting astrocyte function—specifically reducing glutamate clearance and glutamine production. This coincides with decreased brain-derived neurotrophic factor, a protein vital for brain health, ultimately contributing to cognitive issues and depressive symptoms in liver disease patients [35]. Notably, as the primary substrate initiating the urea cycle and a key regulator of nitrogen homeostasis, impaired hepatic processing of ammonia directly disrupts systemic BUN metabolism. This pathophysiological cascade underlies the association between reduced BUN levels and depressive vulnerability identified in our study. Collectively, these findings demonstrate that the liver-brain axis constitutes a critical pathological substrate underlying the development of depressive behaviors. The BUN/Cr ratio serves as a validated biomarker for renal function assessment, reflecting hydration status, protein catabolism, and renal parenchymal injury patterns [36, 37]. Experimental evidence from murine CKD models demonstrates that cerebral oxidative stress, neuroinflammation, and mitochondrial dysfunction correlate with depressive-like phenotypes [38]. In CKD patients, uremic toxin accumulation, particularly tryptophan-derived indoxyl sulfate, exacerbates neuroinflammation and apoptosis through astrocytic dysfunction and blood–brain barrier disruption, thereby elevating the risk of depression [39, 40]. Chronic neuroinflammation coupled with serotonin depletion, both driven by uremic toxin retention, may constitute a central mechanism underlying depression onset and therapeutic resistance in CKD [40]. Beyond classical toxin pathways, recent evidence suggests that glymphatic dysfunction may be a key pathway linking hepatic/renal impairment to mood disorders [41]. This intracranial clearance system, reliant on cerebrospinal-interstitial fluid exchange via perivascular spaces, eliminates neurotoxic proteins (e.g., β-amyloid, tau). In hepatic encephalopathy models, portal hypertension triggers systemic inflammation and cerebrovascular endothelial dysfunction, suppressing AQP4 polarization in astrocytic endfeet and disrupting glymphatic flow. Concurrently, uremic milieu in renal failure impairs AQP4 function, exacerbating toxic protein accumulation [42]. The elevated prevalence of depression in patients with hepatic-renal impairment observed in this study may be closely linked to neuroinflammatory cascades secondary to compromised glymphatic clearance efficiency.

Clinically, these biomarkers enhance depression risk stratification through two pathways. First, concurrent monitoring of ALT/AST ratio, BUN and BUN/Cr ratio provides complementary hepatorenal functional insights, particularly valuable for elderly patients with subclinical organ dysfunction. Second, the linear associations support continuous risk assessment rather than binary classification, enabling dynamic monitoring of depression susceptibility during hospitalization. We propose integrating a depression risk scorecard based on these hepatorenal function biomarkers into the routine admission assessment for geriatric psychiatry patients, enabling rapid "test-and-triage" stratification. For patients with comorbid liver impairment (elevated ALT/AST ratio) or cognitive impairment (e.g., hepatic encephalopathy risk), a low aromatic amino acid dietary regimen may be preemptively designed to reduce pseudo-neurotransmitter accumulation. For those with reduced BUN/Cr ratio or BUN levels, a high-branched-chain amino acid and low-aromatic-amino-acid diet could be implemented to mitigate aromatic amino acid toxicity, thereby improving nitrogen balance. We recommend integrating these biomarkers into existing geriatric depression evaluation protocols and establishing interdisciplinary care pathways involving hepatologists and nephrologists.

Study limitations should be noted. First, the cross-sectional design precludes causal inference between hepatorenal biomarkers and depression, though sensitivity analyses and subgroup analyses supported robustness. Prospective cohorts integrating metabolomics are needed to confirm predictive utility and dissect molecular mechanisms. Furthermore, longitudinal designs are warranted to elucidate the mediating effects of relevant biomarkers (e.g., inflammatory markers) and explore their potential bidirectional relationships with geriatric depression. Second, although covariate adjustment was used to control for partial confounding, residual confounding may persist due to unmonitored dosage changes of other medications (e.g., SSRIs/diuretics) and unmeasured inflammatory markers (e.g., IL-1β), requiring further validation via pharmacological assays and multi-omics analysis in subsequent studies. Third, the single-center sampling from geriatric psychiatry inpatients in Eastern China may introduce selection bias. External validation through multi-center cohorts encompassing diverse healthcare settings is warranted to confirm generalizability. Moreover, this study did not assess the stability of hepatorenal biomarker-depression associations across subtypes or severity levels, thus requiring stratified analyses or longitudinal designs accounting for clinical heterogeneity in future research.

## Conclusion

In conclusion, hepatorenal dysfunction in geriatric psychiatric inpatients shows significant associations with depression risk: elevated ALT/AST ratio and reduced BUN or BUN/Cr ratio levels serve as independent risk factors with early warning potential. These findings highlight the clinical significance of liver-brain and kidney-brain axis interactions in depression pathogenesis, strongly recommending the incorporation of these biomarkers into geriatric depression risk assessment protocols.

## Data Availability

The datasets analyzed during the present study are available from the corresponding author on reasonable request.
